# Discordance between symptom presentation and testing for Clostridioides difficile among hospitalized VA patients

**DOI:** 10.1017/ash.2025.280

**Published:** 2025-09-24

**Authors:** Geneva Wilson, Neill Bates, Ravyn Jackson, Felicia Bixler, Rebecca Cooper, Lishan Cao, Margaret Fitzpatrick, Katie Suda, Charlesnika Evans

**Affiliations:** 1 Edward Hines Jr. VA Hospital; 2 Department of Veterans Affairs; 3 Rebecca Cooper, Edward Hines Jr. VA Hospital; 4 None; 5 University of Colorado Anschutz Medical Center; 6 University of Pittsburgh School of Medicine; 7 Northwestern University Department of Veterans Affairs

## Abstract

**Background:** Presence and documentation of clinical symptoms of Clostridioides difficile infection (CDI) prior to diagnostic testing is not well-described. The Infectious Diseases Society of America (IDSA) guidelines recommend that patients have ≥3 episodes of unexplained loose stool in the previous 24 hours before testing. In populations predisposed to chronic non-infectious diarrhea, such as those undergoing chemotherapy or with chronic gastrointestinal (GI) illness, more explicit signs of infection may be needed. Our objective was to evaluate CDI symptoms that proceeded testing in a cohort of inpatient Veterans with chronic GI illness or undergoing chemotherapy. **Methods:** This retrospective cohort study included Veterans hospitalized at 8 VA facilities from January 1st, 2019-December 31st, 2022, who were tested for CDI, and were receiving chemotherapy or had chronic GI illness. Charts reviewed identified the following symptoms in the 24 hours prior to testing: greater than 3 loose stools in 24 hours, bloody stool, nausea, vomiting, abdominal pain, fever (temperature ≥100.4°F), and white blood cell count >10,000/mm3. The presence of 3 loose stools in 24 hours alone was deemed the minimal indication for CDI testing, while the presence of any additional symptoms was considered high indication for testing. CDI treatment was defined as at least one dose of metronidazole, oral vancomycin, or fidaxomicin ±7 days from testing. Chi-square tests assessed the association between indication for CDI testing and test positivity. **Results:** A total of 676 tests for 577 unique patients were reviewed (69.1% White, 94.5% male, mean age=68.3 years). Most had a chronic GI illness (90%); colitis, and presence of a gastrostomy were the most frequently reported. Only 14% of CDI tests were positive. The minimal indication for CDI testing was present for 243 tests (36%). 190 tests (28%) were ordered for patients with symptoms highly indicative of CDI. Of the negative tests, 55% were associated with at least one dose of CDI treatment. There was no association between test indication and test positivity (p-value=0.82). **Conclusion:** In a population predisposed to chronic non-infectious diarrhea, nearly two thirds (64%) of those tested did not meet the minimum requirement (3 documented loose stools in 24 hours). This may partly explain the low-test positivity rate of 14%. Over half of negative tests were associated with CDI treatment. Future work should focus on diagnostic stewardship to improve documentation of loose stool and other CDI symptoms prior to testing to reduce unnecessary testing and overtreatment.

